# Array of Graphene Variable Capacitors on 100 mm Silicon Wafers for Vibration-Based Applications

**DOI:** 10.3390/membranes12050533

**Published:** 2022-05-19

**Authors:** Millicent N. Gikunda, Ferdinand Harerimana, James M. Mangum, Sumaya Rahman, Joshua P. Thompson, Charles Thomas Harris, Hugh O. H. Churchill, Paul M. Thibado

**Affiliations:** 1 Department of Physics, University of Arkansas, Fayetteville, AR 72701, USA; mngikund@uark.edu (M.N.G.); fharerim@uark.edu (F.H.); jmmangum@uark.edu (J.M.M.); sr082@uark.edu (S.R.); jpt003@uark.edu (J.P.T.); hchurch@uark.edu (H.O.H.C.); 2 Sandia National Laboratories, Albuquerque, NM 87123, USA; ctharri@sandia.gov

**Keywords:** graphene, photolithography, wet etching, variable capacitor, atomic force microscopy, critical point drying, graphene transfer, integrated circuit

## Abstract

Highly flexible, electrically conductive freestanding graphene membranes hold great promise for vibration-based applications. This study focuses on their integration into mainstream semiconductor manufacturing methods. We designed a two-mask lithography process that creates an array of freestanding graphene-based variable capacitors on 100 mm silicon wafers. The first mask forms long trenches terminated by square wells featuring cone-shaped tips at their centers. The second mask fabricates metal traces from each tip to its contact pad along the trench and a second contact pad opposite the square well. A graphene membrane is then suspended over the square well to form a variable capacitor. The same capacitor structures were also built on 5 mm by 5 mm bare dies containing an integrated circuit underneath. We used atomic force microscopy, optical microscopy, and capacitance measurements in time to characterize the samples.

## 1. Introduction

Developing technologies related to clean energy, harvesting energy, and the conversion and storage of energy has become a high priority in recent years [1,2,3]. Energy sources such as ambient vibrations hold the potential for battery alternatives in certain applications. Continued device miniaturization is driving this need. Consequently, extensive research in devising on-board energy harvesting power sources for ultra-low power integrated circuits is underway [4,5,6,7].

Flexible suspended membranes have enabled a wide range of technologies, from acoustic wave generators and receivers [8,9,10,11] to nanoelectromechanical sensors [12,13]. One of the most exciting membranes is graphene. Graphene membranes may improve these applications as they exhibit exceptional physical properties, including high electrical conductivity, high thermal conductivity, unusual flexibility, enormous specific surface area, and remarkable biocompatibility [14,15,16,17,18,19,20]. Consequently, graphene membranes may enhance performance in applications such as supercapacitors [21,22], photovoltaic devices [23], nanoelectromechanical [24], and micro-electromechanical devices [25,26].

For example, single-layer graphene was suspended over a lightweight, flexible, transparent, and conductive bilayer composite of polyetherimide to create an energy-efficient, high-performance nanoelectromechanical transducer [27]. Graphene membranes have also been used to make a graphene varactor, allowing its capacitance to be finely tuned using voltage-induced deflection [28,29].

Freestanding graphene membranes also show promise as a source of vibrational energy for low-power electronics [30,31,32,33,34]. A device based on strained nanostructured graphene has been proposed for vibration energy harvesting from thermal fluctuations and ambient vibrations [35].

**Figure 1:**
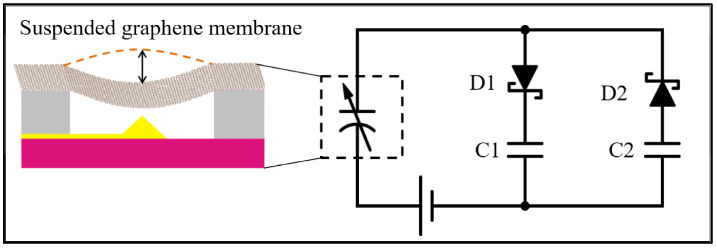
Schematic of graphene energy harvesting circuit using two Schottky diodes, two storage capacitors, a variable capacitor, and a DC bias voltage. The variable capacitor represents the suspended graphene over a fixed metal electrode, as shown in the side illustration.

A graphene-based variable capacitor connected to an energy harvesting circuit is shown as a schematic in Figure 1. In this implementation, the upper capacitor plate is flexible, replacing the usual rigid plate held by springs [36,37,38]. Using springs works well but generally leads to a larger overall device, as shown in Table 1.

The graphene is suspended above a fixed metal electrode to form the variable capacitor. As the graphene membrane vibrates up and down, the distance between graphene and electrode changes, which causes the capacitance to change in time. As the capacitance varies in time for a fixed voltage, electrical charge is forced on and off the graphene membrane. This comes from the equation q(t) = C(t) V, where q is the charge, C is the varying capacitance, and V is the bias voltage. When the charge flows clockwise, it passes through diode D1 and charges capacitor C1. When the charge flows counterclockwise, it moves against the bias voltage V, charges capacitor C2, then passes through diode D2. If the bias voltage source is a rechargeable battery, it will be charged during this half-cycle [39,40].

Even at a current level of nanoamps, this circuit was found to be 50% efficient when operated at its maximum output power [41]. A recent scanning tunneling microscopy (STM) study used a similar circuit to monitor the current driven by the ambient motion of freestanding graphene by fixing the location of the STM tip (feedback off mode). These findings provided the motivation for this work [42].

For this study, we fabricated an array of freestanding graphene variable capacitors on 100 mm silicon wafers. We used standard silicon fabrication processes, along with other well-established techniques developed by the semiconductor industry [43,44,45,46,47,48,49,50,51]. However, our graphene-well structure has requirements that make its development unique, and we share the process here. The fabrication of the graphene variable capacitor array was reduced to a three-step process. First, the trench, well, and tip features were created by etching an underlying sacrificial silicon dioxide layer [44,52,53,54]. Second, the metal traces and bonding pads were deposited. Finally, the large-area graphene transfer and suspension were achieved using a critical point dryer.

## 2. Materials and Methods

**Figure 2:**
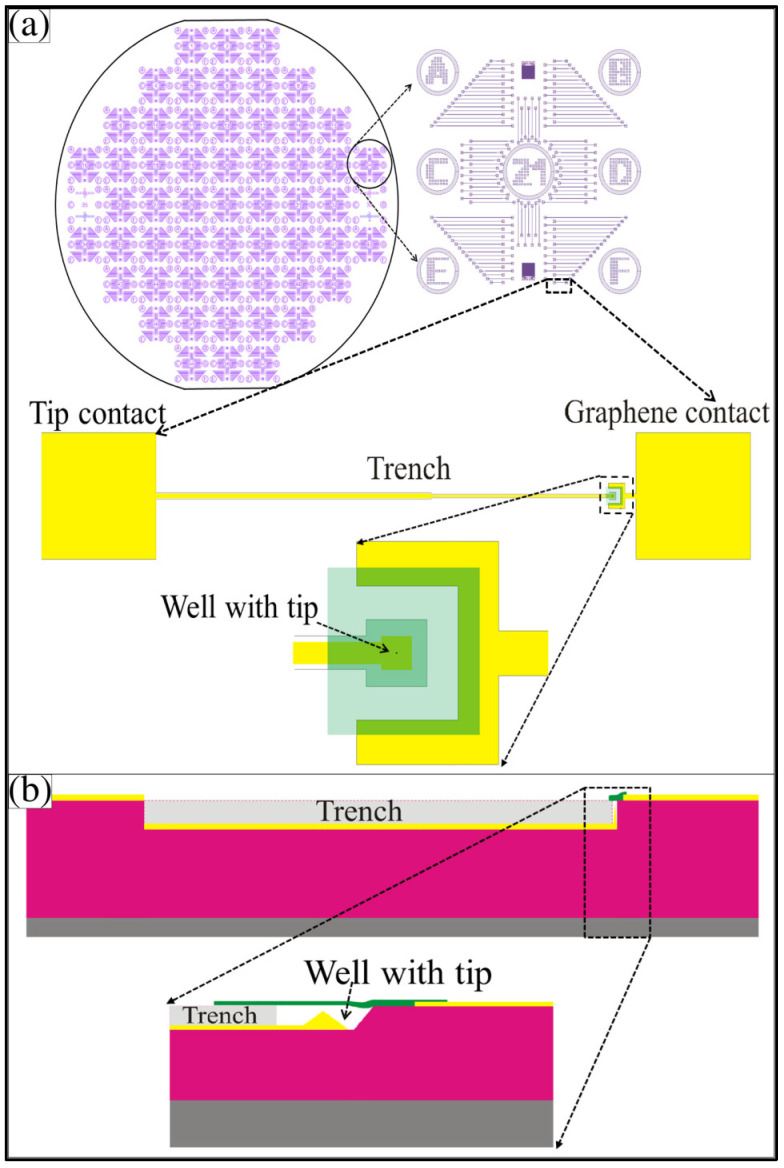
Schematic illustration of the wafer layout. (**a**) 100 mm silicon wafer with an array of 57 identical patterns next to a zoomed-in view of a single 1 cm square unit. Notice each 1 cm unit has regions A-F labeled for easy record keeping. A top view illustration of a single graphene membrane variable capacitor with metal contacts is also shown. (**b**) Cross-sectional side view and a zoomed-in side view of a single graphene membrane variable capacitor.

For this study, we used low resistivity (1–10 Ω cm), 500-µm-thick, 〈100〉-oriented silicon wafers with a 2-µm-thick, high-quality thermal oxide on top (University Wafer, Boston, MA, USA). A final design concept for a completed wafer containing thousands of graphene membrane variable capacitors is illustrated in Figure 2. The pattern was aligned to the wafer cleavage planes. It had 57 identical 1 cm by 1 cm structures arranged in a square grid, as shown in Figure 2a. Each unit was numbered. A zoomed-in view of unit 21 is shown, which had more than 100 graphene membrane variable capacitor test structures with varying trench lengths. The shortest trench length in section F is shown, with an expanded view just below unit 21. Each variable capacitor had two metal contacts (tip and graphene membrane) separated by a trench to help avoid electrical shorts. In the top view, the two metal contacts are shown. The tip-well structure was on the right, with a zoomed-in view below. The semi-transparent green sheet is graphene, which covers the square well and touches the metal graphene membrane contact pad to its right. A metal trace ran from the tip along the bottom of the trench to the metal pad on the far left. The trench provided space for the placement of the graphene membrane while avoiding contact with the left metal contact, which connected to the tip. A cross-sectional side view of the single variable capacitor is shown in Figure 2b. A zoomed-in view of the graphene membrane suspended over the square well (with the metal cone underneath) is also shown. The zoomed-in side view helps illustrate how the capacitance of the structure varies in time as the graphene moves.

A buffered oxide etchant (5:1 with hydrofluoric acid) (VWR, Suwanee, GA, USA) cut the trench-well-tip structures into the silicon dioxide. Atomic force microscopy (AFM) (Mnp, Easton, PA, USA) and optical microscopy (Olympus, Pittsburgh, PA, USA) were used to characterize the etched surface morphology and the top of the wafer. The conductive paths and contact pads were created by depositing a thin Cr layer (5 nm), followed by a thicker Au layer (50–100 nm). AFM and optical microscopy were used to characterize these layers. Graphene, commercially grown on Ni, was used for the large-area graphene membrane transfer (graphene supermarket). A critical point dryer was used to aid in graphene membrane suspension.

## 3. Results and Discussion

### 3.1. Mask 1 Processes: Trench, Well, and Tip

**Figure 3:**
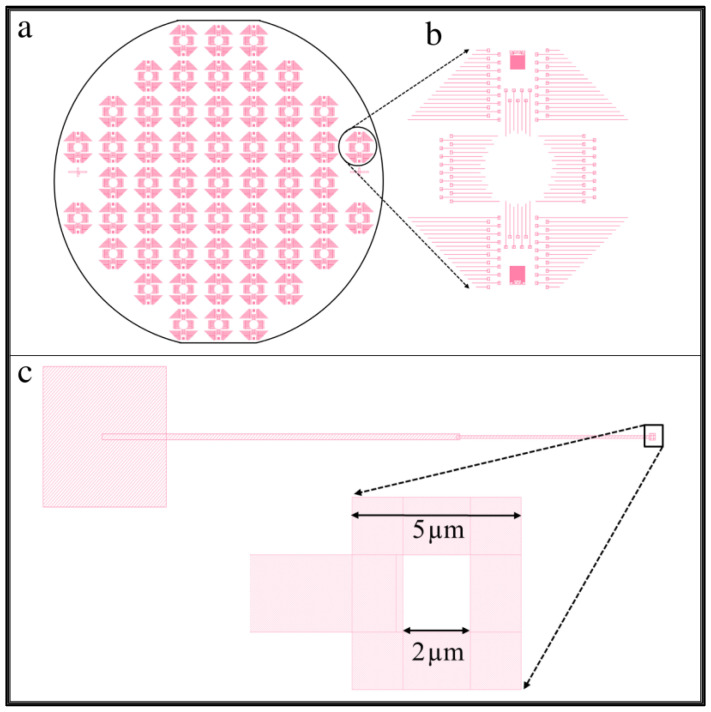
Mask 1 design pattern. (**a**) Fully patterned wafer with an array of 57 identical square sections. (**b**) Zoomed-in view of a 1 cm × 1 cm region. (**c**) A pattern that will yield a single trench, well, and tip structure.

Mask 1 defines the position and size of the trench, well, and tip features, as shown in Figure 3. It allows a full 100 mm silicon wafer to be patterned all at once, creating 57 identical regions measuring 1 cm by 1 cm each. The edges of the square regions were oriented along the cleavage planes, as shown in Figure 3a. A zoomed-in view of one square unit is shown in Figure 3b, containing more than 100 patterns that ultimately give rise to the long trench, the square well, and the tip. A zoomed-in view of one individual pattern is shown in Figure 3c.

The shaded areas of the pattern were etched, while the white areas were protected from the etchant. We designed a square-shaped ring at the right end of the trench, shown in a zoomed-in view at the bottom of Figure 3c. Within the 5 µm by 5 µm shaded region is a 2 µm by 2 µm unshaded area. This pattern allowed the square well to form with a cone-shaped tip at its center. The process etches isotropically, so the shaded section and the area underneath the 2 µm by 2 µm protected region were etched simultaneously. In time, the etchant fully undercut the central region, causing the protective resist to float away. At the end of the etching process, a cone-shaped tip remained in the center of the square-well structure.

**Figure 4:**
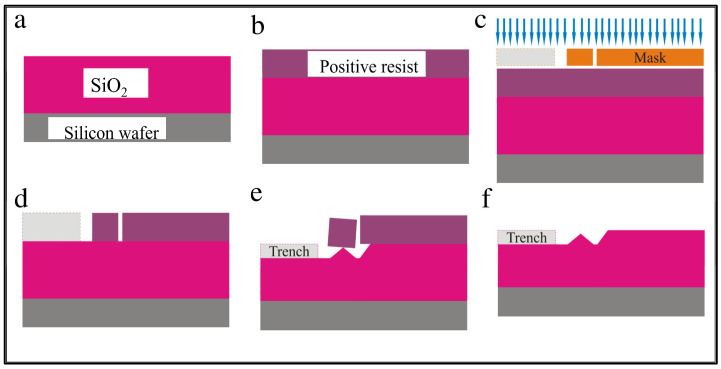
Schematic illustration of trench, well and tip structure formation processing steps. (**a**) Low resistivity silicon wafer with a 2-µm-thick SiO_2_ layer; (**b**) resist layer spin-coated on wafer and baked for 5 min; (**c**) Mask 1 is applied during lithography exposure; (**d**) patterned wafer after developing; (**e**) isotropic etching of SiO_2_ layer in 5-min hydrofluoric acid bath for trench, well, and tip formation; and (**f**) final wafer with trench, well, and tip feature after resist is removed.

The steps for creating the trench, well, and tip feature using lithography are illustrated in Figure 4. The overall idea is as follows. On top of the silicon wafer is a two-micron-thick silicon dioxide layer. Using a stencil, we etched a pattern into the silicon dioxide using hydrofluoric acid. With this acid, the silicon dioxide etches isotropically, so it is partially removed underneath the stencil as well as straight down toward the silicon wafer. When the etching was complete, a three-dimensional trench was formed in the insulating silicon dioxide layer.

First, a layer of photoresist AZ 5214 (MicroChemicals, St. Louis, MO, USA) E or e-beam resist polymethyl methacrylate (PMMA) was spin-coated on the SiO_2_ layer and baked at 180 °C for 5 min. Then, the lithographic Mask 1 was used during the exposure process to mark the pattern on the wafer. Once exposed, the pattern was developed for 30 s in n-amyl acetate, then 20 s in methyl isobutyl ketone (MIBK) (Fisher Scientific, Waltham, MA, USA), and finally rinsed with isopropyl alcohol (IPA) (Fisher Scientific, Waltham, MA, USA) to remove the exposed resist.

The wafer was then placed in a 5:1 buffered oxide etch containing hydrofluoric acid (HF) for 5 min at room temperature. This step etches the exposed SiO_2_ material isotropically and undercuts the resist to form a tip, as illustrated in Figure 4e. The etch time determines the depth of the trench and the height of the tip structure. The wafer was then double rinsed in deionized water to stop any further etching. Finally, the sample was soaked overnight in Remover PG to eliminate the photoresist, rinsed with IPA, then blown dry with nitrogen to keep the wafer spot-free.

**Figure 5:**
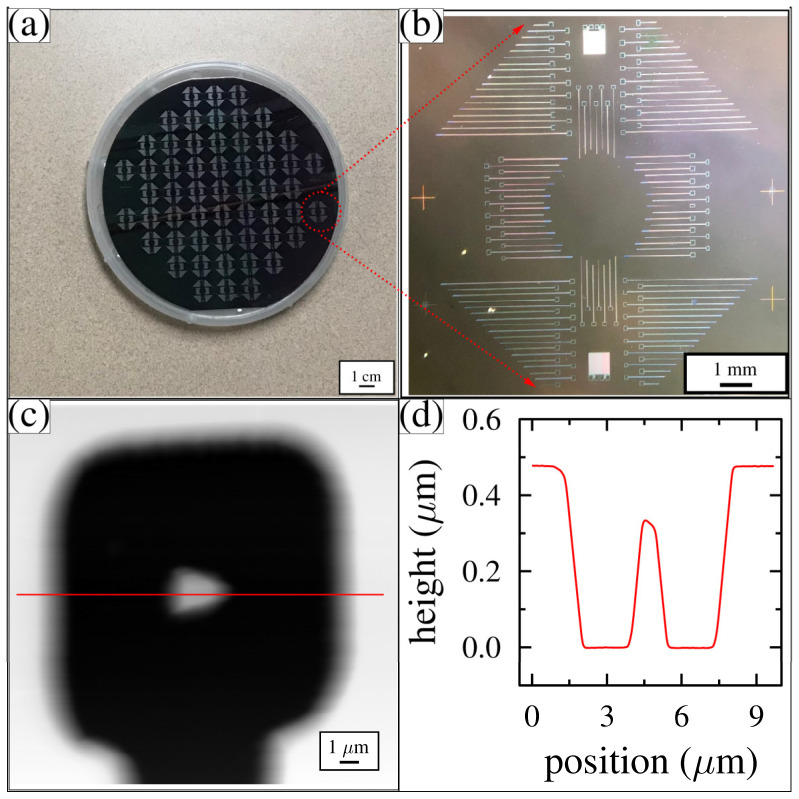
Optical microscopy and AFM images after Mask 1. (**a**) Patterned 100 mm wafer with an array of 57 identical features; (**b**) high resolution optical image of a single 1 cm square unit; (**c**) AFM image of a single square well with a cone-shaped tip at its center; and (**d**) AFM line profile taken horizontally through the AFM image shown in (**c**).

To confirm if the wafer was successfully etched and the resist completely removed, optical microscope images were taken, as shown in Figure 5a,b. The pattern was transferred to the wafer, uniformly covering the entire surface. Next, AFM was used to measure the topography of various tip-well structures throughout the wafer. A typical AFM image and its cross-sectional line profile are shown in Figure 5c,d. The top of the wafer is white. The square well is mostly black, with the tip appearing as a white central feature. The trench intersects the well along its bottom edge. In the cross-sectional line profile, which cuts horizontally through the center of the AFM image, the 0.5 μm-deep well can be seen. A tip protrudes from the center of the bottom of the well. The highest point of the tip is 0.15 μm from the top of the wafer. This pattern was reproduced with a high degree of accuracy across the entire wafer.

### 3.2. Mask 2 Processes: Metal Traces and Bonding Pads

**Figure 6:**
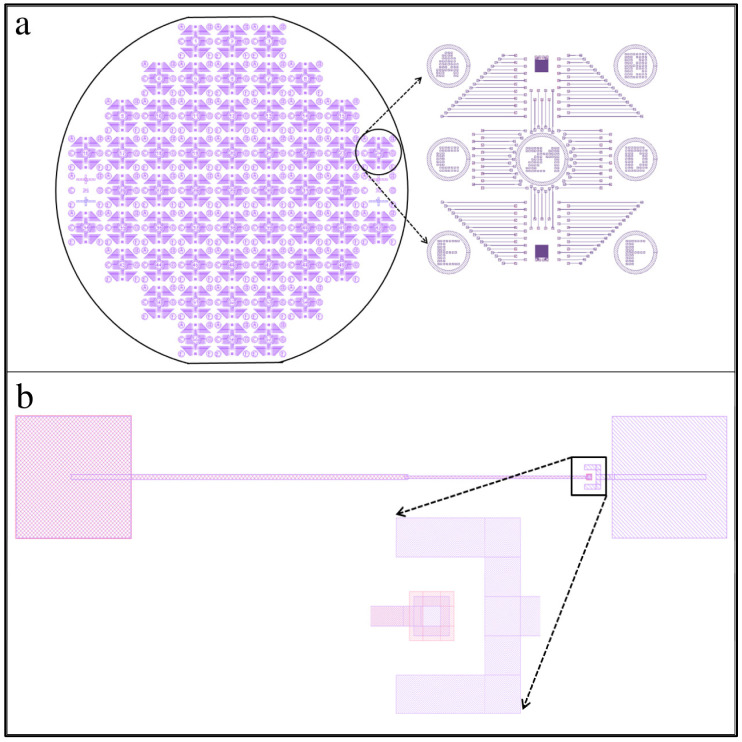
Mask 2 design pattern. (**a**) Fully patterned wafer with 57 identical units and a zoomed-in view of a single 1 cm unit. Notice each 1 cm unit is numbered (this one is unit 21) and has regions A-F labeled for easy record keeping.; and (**b**) A single trench, well, and tip structure with a zoomed-in view of the tip-well region with a graphene membrane bonding pad to the right of the tip-well structure.

Mask 2 defines the position and size of the conductive metal traces and electrical contact pads. The full wafer mask design is shown in Figure 6a. It must be precisely aligned to Mask 1. To aid the alignment, several crosshair markers were written into the wafer during the Mask 1 etch, which can be seen in both Figure 3a and Figure 5b. A zoomed-in view of a single 1 cm square region for the Mask 2 design is shown to the right in Figure 6a. This mask also writes the labels into each unit (the number 21 can be seen). A zoomed-in view of a single trench-well-tip structure is shown in Figure 6b, which also contains another zoomed-in view of the tip-well region. A metal contact pad (smaller than the square well but larger than the tip) was used inside the square well to coat the tip and to ensure the tip was coated with metal. In addition to a metal contact pad to the right of the tip-well region, we also added a metal horseshoe-type design around the tip-well structure. This was placed on the top of the wafer and connected to the graphene membrane contact pad. The horseshoe shape ensures electrical contact with the graphene membrane was made when the graphene membrane was placed over the well at a later step in the fabrication process.

**Figure 7:**
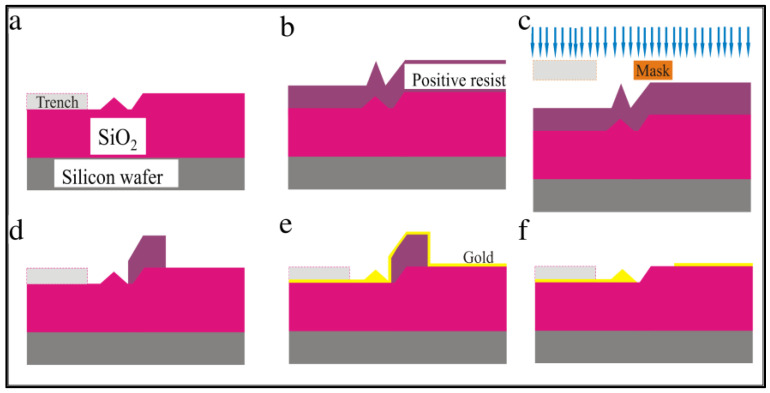
Schematic illustration of Mask 2 processes. (**a**) Wafer after Mask 1 showing the trench-well-tip features throughout. (**b**) Resist layer spin-coated and baked for 5 min. (**c**) Mask 2 is applied during lithographic exposure. (**d**) Patterned device after developing. (**e**) Deposition of 100 nm of gold. (**f**) Final metallized wafer after liftoff.

The Mask 2 processing steps are illustrated in Figure 7. First, either AZ 5214E (MicroChemicals, St. Louis, MO, USA) or α-methyl styrene-co-α-chloroacrylate methylester (CSAR) (Sigma-Aldrich, St. Louis, MO, USA) resist layer was spin-coated and baked at 180 °C for 5 min. Mask 2 was then used to pattern the resist. Next, the wafer was developed for 30 s in n-amyl acetate, 20 s in methyl isobutyl ketone (MIBK), and rinsed in IPA. Then 5 nm of chromium was deposited onto the surface, followed by 100 nm of gold. The thickness of the gold layer is a variable parameter used to control the distance between the top of the wafer and the top of the tip. The wafer was then soaked overnight in a 50-50 acetone-remover PG mixture for liftoff, followed by an IPA rinse. Finally, the sample was blown dry with nitrogen gas to avoid spotting.

**Figure 8:**
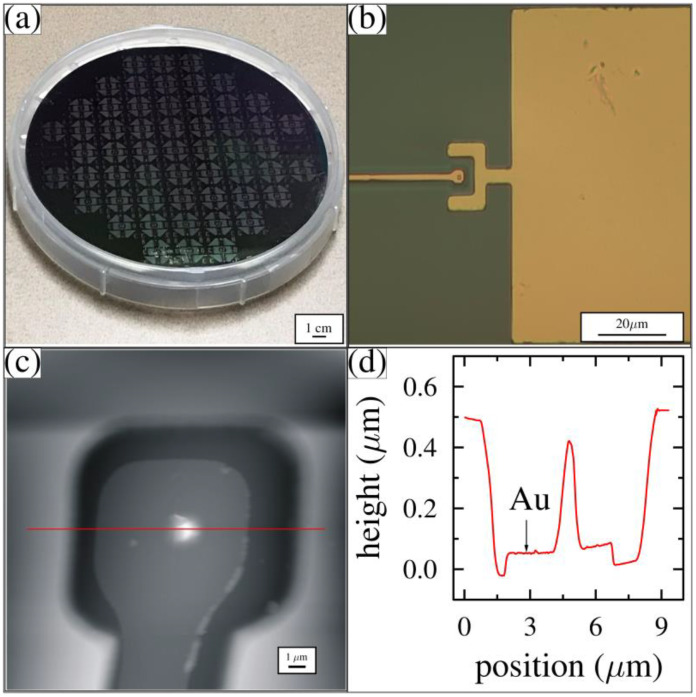
Optical and AFM images. (**a**) Mask 2 patterned full wafer; (**b**) tip-well feature with bonding pad after gold deposition; (**c**) AFM image of a single tip-well structure; and (**d**) horizontal line profile through the tip in (**c**) that reveals the gold thickness.

Optical microscopy and AFM images were used to characterize the wafer after completing the Mask 2 processing steps. An optical photograph of the full wafer is shown in Figure 8a. The pattern was reproduced across the entire wafer. A high-resolution image of the gold-coated tip, trench, and contact pad around the well structure is shown in Figure 8b. The horseshoe spans three sides of the tip-well structure. AFM was used to characterize the position of the metal deposited inside the well and to determine the thickness of the metal layer, as shown in Figure 8c,d. From this image, we confirmed both the thickness of the gold and the distance between the top of the tip and the top of the wafer. This pattern was reproduced with a high degree of accuracy across the entire wafer.

### 3.3. Graphene Membrane Placement and Suspension

**Figure 9:**
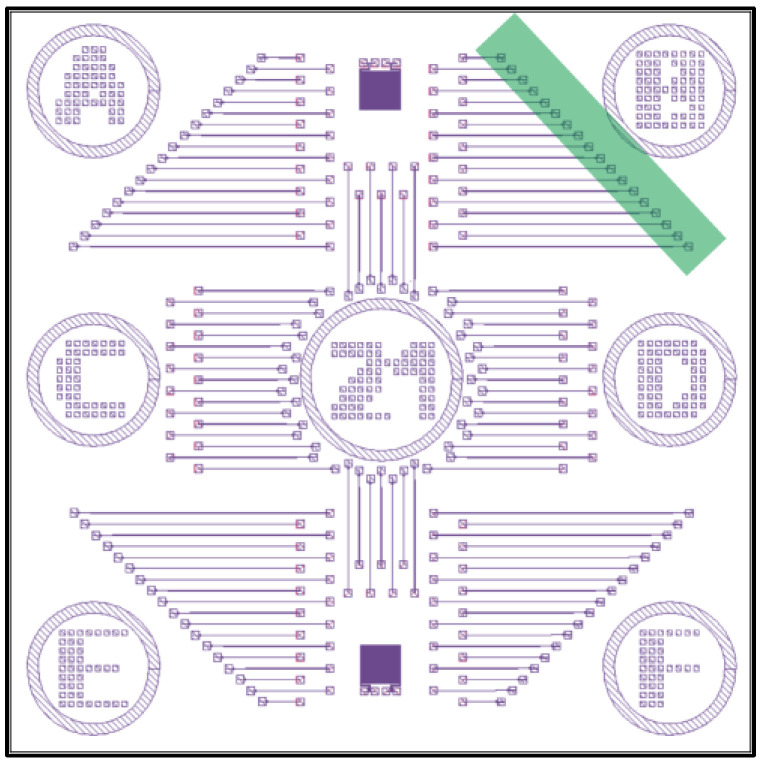
Illustration of graphene membrane placement on the wafer.

We used commercially available multilayer graphene grown on nickel on a silicon chip (graphene supermarket, Ronkonkoma, NY, USA). This stage of the process ensures that the graphene membrane covers the well over the tip, that it makes electrical contact with the graphene membrane contact pad, and that it does not make electrical contact with the tip contact pad. The graphene membrane is suspended over the array of tip features and does not contact any of them. The shape of the graphene membrane and its placement location for region B on unit 21 of the wafer is shown schematically as a rectangular green strip in the upper right corner of Figure 9.

**Figure 10:**
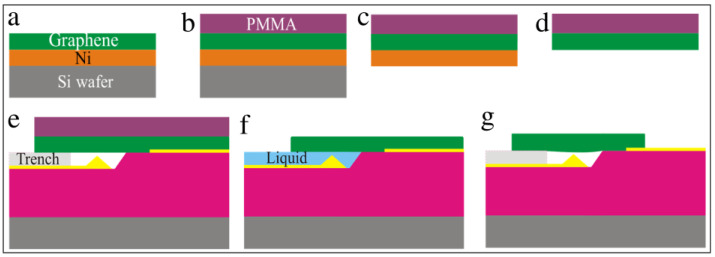
Graphene transfer steps. (**a**) Graphene-Ni-Silicon chip. (**b**) Spin coat a PMMA layer. (**c**) Peel away the PMMA-graphene-Ni layer from the silicon with tape. (**d**) Etch away the Ni layer. (**e**) Place the PMMA-graphene on patterned wafer over an array of tip-well structures. (**f**) Apply hexane, followed by critical point drying. (**g**) Final graphene suspended over the tip.

The large-area graphene membrane transfer process is schematically illustrated in Figure 10 [55,56,57]. Using a commercially prepared graphene-Ni-Silicon chip stack, we spun-coated a layer of PMMA over the graphene and baked it at 180 °C for 4 min. Next, Scotch tape was used to peel off a thin strip of the PMMA-graphene-Ni layer from the silicon chip. The strip was placed in an FeCl_3_ etchant for 20 min to dissolve the nickel. The tape-PMMA-graphene stack was transferred to DI water to remove the FeCl_3_ solution. The tape was then removed from the PMMA-graphene stack, and the graphene membrane was placed on the desired region of the wafer. The entire wafer with the PMMA-graphene was then set at an angle for 10 min to dry in position (not shown). The PMMA was later removed by soaking the sample in acetone for 10 min. The sample was then quickly transferred to hexane. The liquid hexane was not allowed to air dry. Instead, the entire system was introduced into a critical point dryer (CPD), which dried the graphene-wafer system and assisted in the suspension of the graphene membrane over the tip features.

**Figure 11:**
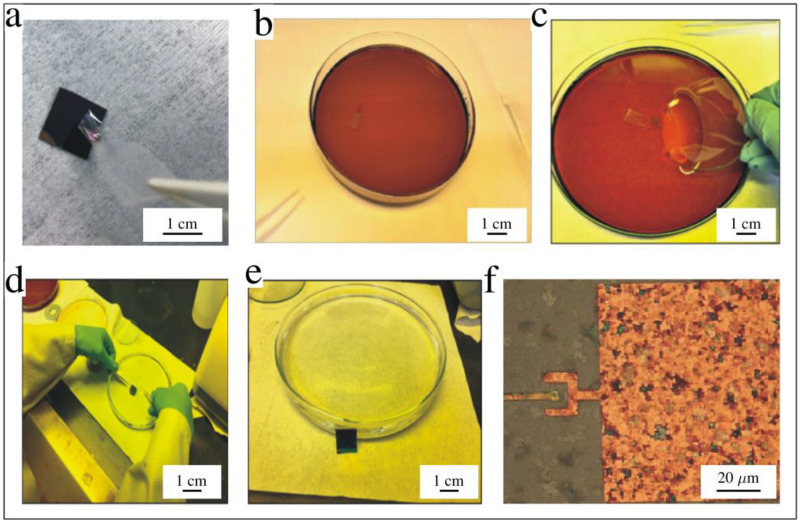
Images of graphene transfer process: (**a**) tape used to peel off a thin strip of Ni-graphene-PMMA layer; (**b**) nickel-graphene-PMMA layer in FeCl_3_ solution to etch off nickel; (**c**) watch glass used for scooping graphene; (**d**) aligning graphene to the tip regions of the device; (**e**) placing the sample at an angle for graphene-PMMA layer to stick to the sample; and (**f**) optical image showing graphene location on the device.

The photographs of the various graphene transfer steps are shown in Figure 11. Tape was used to peel off the PMMA-graphene-Ni layer from the silicon chip. The Ni layer was etched in FeCl_3_. The tape-PMMA-graphene was scooped out of the etchant, placed on our patterned wafer, and then set at an angle to dry. After using hexane and CPD, the graphene membrane was suspended over the tip well structures, as shown in the optical image in Figure 11f. Note that the size of the suspended graphene is too large for us to suspend single-layer graphene. Thus, optical characterization was sufficient to ensure proper graphene placement.

### 3.4. Processing Bare Die with a Subsurface Integrated Circuit

**Figure 12:**
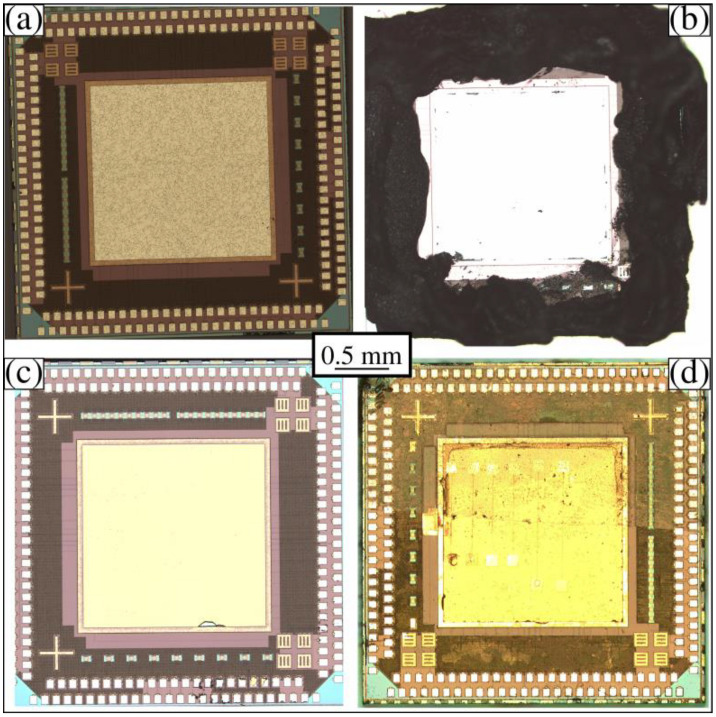
TSMC chip procedure. (**a**) Chip obtained from TSMC. (**b**) Chip with the outer bonding pads covered with carbon paint. (**c**) Chip after etching away the central aluminum bonding pad. (**d**) Graphene membrane suspended over multiple trench-well-tip features.

In addition to developing the graphene membrane variable capacitor fabrication process on 100 mm silicon wafers, we also reproduced the process on a bare die containing an integrated circuit built by the Taiwan Semiconductor Manufacturing Corporation (TSMC) (MUSE Semiconductor, Denver, CO, USA). The TSMC chip measures 5 mm by 5 mm and contains a rectifier amplifier circuit for energy harvesting. The top of the TSMC chip has bonding pads around the perimeter, as well as a 3 mm by 3 mm bonding pad in the center. The bonding pads are aluminum and are shown in Figure 12a. To remove the central aluminum, we first covered the outer regions of the TSMC chip with carbon paint, as shown in Figure 12b. Next, the TSMC chip was etched in a commercial aluminum etchant (Transene) (VWR, Suwanee, GA, USA) for 20 min, followed by a DI water double rinse. Figure 12c shows the chip after etching. Next, we deposited SiO_2_ over the TSMC chip at low temperatures to avoid altering the integrated circuit. The TSMC chips were then run through the same three-step process discussed above. After Mask 1, Mask 2, and graphene membrane placement, an optical image of the TSMC chip was taken, as shown in Figure 12d. The graphene membrane was semi-transparent and covered the entire upper half of the chip. A line running nearly horizontally across the chip marks the lower edge of the graphene membrane. During the metal deposition steps, the graphene membrane was wired automatically to the top bonding pads, and the tips were wired to the lower bonding pads.

### 3.5. Capacitance Characterization

**Figure 13:**
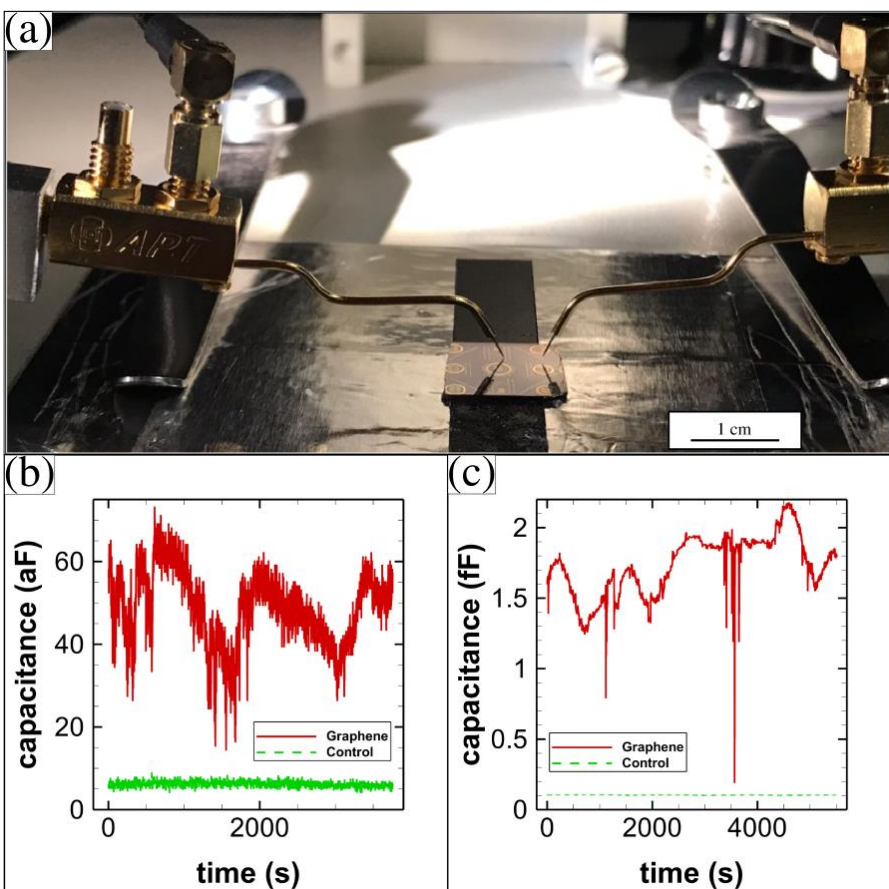
Capacitance measurements. (**a**) Photograph of measurement probes on the wafer; (**b**) capacitance in time for the capacitors made using the 100 mm silicon wafers; and (**c**) capacitance in time for the capacitors made using the TSMC bare die.

We used a precision high-resolution capacitance meter (AH 2550A) (Andeen-Hagerling, Cleveland, OH, USA) to measure the capacitance between the graphene membrane metal contact pad and the tip metal contact pad. A photograph of our setup is shown in Figure 13a. We used shielded measurement probes to touch both the graphene membrane metal contact and the tip metal contact. The setup was completely grounded and was inside a dark box to avoid interference from its surroundings. We first determined if the graphene membrane directly contacted the tip by measuring the resistance between the two probes. Generally, when properly suspended, the resistance was found to be much higher than 10 GΩ. The average resistance was 2.039 GΩ for the TSMC bare die sample and 128.576 GΩ for the 100 mm silicon wafer sample.

The capacitance varied in time for the graphene-membrane variable capacitors that were fabricated on the 100 mm wafers. A typical result is shown in Figure 13b. The variation in capacitance was about 60 aF for the graphene membrane capacitor (a constant capacitance was subtracted from the raw data). A control is also shown that has a much smaller variation. The control measurement was made by first raising the probes vertically off of the contact pads until the probes no longer contacted the surface, then repeating the capacitance measurement in time. A typical capacitance measurement for a sample fabricated on the TSMC bare die is shown in Figure 13c. The variation in capacitance was about 2 fF for this sample. We do not attribute the slight drift in capacitance over the 1.5-h long measurement to anything. A control for this sample is also shown.

## 4. Conclusions

We described the design and fabrication of an array of graphene-based variable capacitors on both 100 mm silicon wafers and bare dies manufactured by TSMC. We provided a template for building a massive array of membrane devices in parallel with nearly 10,000 individually addressable membrane suspensions on one substrate. Our procedure is easy to follow and requires only two masks to complete. Photolithography or e-beam lithography can be used to pattern the wafers. Trench-well-tip features were created using an undercutting isotropic wet etch of SiO_2_ with buffered HF for 5 min at room temperature. The conductive pathways and contact pads were created using the patterned deposition of gold. We performed large-area graphene membrane transfer over the tip regions. We used a critical point dryer to ensure that the graphene membrane was left freestanding over the tip feature. The graphene-tip feature forms a variable capacitor with the graphene membrane as the movable plate. Capacitance and resistance measurements taken from these devices confirmed that the graphene membrane was freestanding over the tip structure and was always moving. In the future, a new TSMC chip will be designed. We imagine it will measure 5 mm by 5 mm, have a central region for graphene that measures 3 mm by 3 mm, and contain 100 graphene variable capacitors. Overlaying the graphene on the chip will automatically connect it to the rectifying circuit below. As the graphene is driven by the ambient environment, the storage capacitors will be charged. A typical low-power application would be a battery replacement for reading a remote sensor and transmitting its value to another location.

## Figures and Tables

**Table 1:** Comparisons of key features for variable capacitance device structures.

Vibrating Material	Thickness	Overall Size	Number of Caps	Reference
Silicon	512 microns	15 mm	1	[36]
Polymer	450 microns	11 mm	1	[37]
Spring Steel	1270 microns	66 mm	1	[38]
Graphene	0.001 microns	5 mm	100	This Study

## Data Availability

The data that support the findings in this study are available from the corresponding author upon reasonable request.
